# Systematic review and meta-analysis of the predictive value of C-reactive protein in postoperative infections

**DOI:** 10.1186/1753-6561-5-S6-P29

**Published:** 2011-06-29

**Authors:** BK Nunes, RA Lacerda, JM Jardim

**Affiliations:** 1 University of São Paulo, São Paulo, Brazil

## Introduction / objectives

Systematic review and meta-analysis on C-reactive protein (CRP) to determine its value in predicting prognosis / diagnosis of infection in surgical patients.

## Methods

The sources were searched: Cochrane, Embase, Lilacs, Pubmed / Medline, Ovid, and references of studies found. Was used the PICO strategy for the definition of descriptors. The data of the studies were obtained through a systematic instrument, and the strength of evidence was classified according to the *Center for Evidence Based Medicine*. For the statistical analysis software was used Meta- Disc version beta 1.1.1 (freeware).

## Results

20 studies were included, 18 classified with the highest level of evidence. All reported elevated levels of CRP after surgery and in the presence of postoperative infections (PO), 8 studies a peak CRP between the 2nd and 3rd postoperative day was reported as a normal curve of CRP declined for patients without complications postoperatively, and rising in patients with complications. In four studies it was observed that a value greater than 5 mg /dl in the postoperative CRP is indicative of infection. And the patients with levels above 140 mg/dl in the 4th OP are more likely to develop infections. The meta-analysis revealed a mean of 85% (sensitivity), 86% (specificity), the area under the SROC curve was 0.9060, and odds ratio was 23.56.

## Conclusion

CRP, together with other clinical interventions, has high value in the prognosis / diagnosis in the development of postoperative infection.

## Disclosure of interest

None declared.

